# Potential of Chitosan and Its Derivatives for Biomedical Applications in the Central Nervous System

**DOI:** 10.3389/fbioe.2020.00389

**Published:** 2020-05-05

**Authors:** Doddy Denise Ojeda-Hernández, Alejandro A. Canales-Aguirre, Jorge Matias-Guiu, Ulises Gomez-Pinedo, Juan C. Mateos-Díaz

**Affiliations:** ^1^Biotecnología Industrial, CONACYT Centro de Investigación y Asistencia en Tecnología y Diseño del Estado de Jalisco (CIATEJ), Zapopan, Mexico; ^2^Unidad de Evaluación Preclínica, Biotecnología Médica y Farmacéutica, CONACYT Centro de Investigación y Asistencia en Tecnología y Diseño del Estado de Jalisco (CIATEJ), Guadalajara, Mexico; ^3^Servicio de Neurología, Instituto de Neurociencias, Instituto de Investigación Sanitaria San Carlos (IdISSC), Hospital Clínico San Carlos, Madrid, Spain

**Keywords:** chitosan, chitosan derivatives, central nervous system, drug delivery, tissue engineering, regenerative medicine

## Abstract

It is well known that the central nervous system (CNS) has a limited regenerative capacity and that many therapeutic molecules cannot cross the blood brain barrier (BBB). The use of biomaterials has emerged as an alternative to overcome these limitations. For many years, biomedical applications of chitosan have been studied due to its remarkable biological properties, biocompatibility, and high versatility. Moreover, the interest in this biomaterial for CNS biomedical implementation has increased because of its ability to cross the BBB, mucoadhesiveness, and hydrogel formation capacity. Several chitosan-based biomaterials have been applied with promising results as drug, cell and gene delivery vehicles. Moreover, their capacity to form porous scaffolds and to bear cells and biomolecules has offered a way to achieve neural regeneration. Therefore, this review aims to bring together recent works that highlight the potential of chitosan and its derivatives as adequate biomaterials for applications directed toward the CNS. First, an overview of chitosan and its derivatives is provided with an emphasis on the properties that favor different applications. Second, a compilation of works that employ chitosan-based biomaterials for drug delivery, gene therapy, tissue engineering, and regenerative medicine in the CNS is presented. Finally, the most interesting trends and future perspectives of chitosan and its derivatives applications in the CNS are shown.

## Introduction

The central nervous system (CNS) consists of the brain, spinal cord, and retina, which are composed of more than 100 billion individual nerve cells surrounded by bone structures (Payne et al., 2019). The CNS has long been recognized as immune-privileged, attributed to the blood brain barrier (BBB) and the lack of lymphatic vessels within the parenchyma (Engelhardt et al., 2016). Nevertheless, the CNS is unable to generate robust adaptive immune responses (Ransohoff and Brown, 2012). In the absence of immediate or long-term medical care, this situation could lead to permanent damage or death following a severe nervous system injury (Weil et al., 2008). The treatment of CNS diseases gets further complicated by the BBB, which acts as a shield for foreign substances including therapeutic molecules (Huang et al., 2017). Potential treatments against neurodegenerative disorders are considered difficult to implement because of the limited access to the CNS and the aggressiveness of surgical interventions (Tysseling and Kessler, 2017).

It is now known that central axons are capable of regenerating after injury, but their success is highly dependent on their local environment (He and Jin, 2016; Tedeschi and Bradke, 2017). The composition of the microenvironment is defined by the presence of reactive neural cells. Astrocytes and microglia secrete biomolecules as cytokines, chemokines and growth factors in response to insults. These cells display a big heterogeneity (in morphology, function, and gene expression) and have been associated with both beneficial and detrimental regenerative outcomes on CNS injury (Anderson et al., 2014; Karve et al., 2015). It is also known that the adult CNS possesses neural stem cells with the ability to differentiate into neurons and glia. However, these stem cells need a neurogenic microenvironment to achieve migration and differentiation (Gáge and Temple, 2013). Recent advances in biomaterials have encouraged the search to overcome these challenges, either on their own or as vehicles for stem cell, genetic material, or bioactive molecule delivery (Führmann and Shoichet, 2018). These biomaterials can have a natural or synthetic origin. Natural biomaterials often present good biocompatibility, biodegradability, and cell adhesion but can exhibit some disadvantages as poor mechanical properties or trigger an immune response. Their synthetic counterparts are often easier to chemically modify and have low immune responses but may contain toxic substances (Lim and Spector, 2017; Wang Y. et al., 2018). Thus, natural and synthetic biomaterials are frequently used together to exploit the advantages of both, resulting in products with the desired characteristics for each application.

Currently, chitosan is one of the leading natural biomaterials for CNS applications, both in its natural form or as a modified derivative. In biomolecules delivery, it stands out for its penetration enhancement ability and mucoadhesive capacity, which make it a great material for nose-to-brain approaches (Rassu et al., 2016; Yu et al., 2019). In tissue engineering and regenerative medicine, chitosan and its derivatives have shown to promote axonal regeneration, anti-inflammation, and to successfully deliver neurotrophic factors and cells with a consequently functional recovery (Wang Y. et al., 2018). In this way, chitosan-based biomaterials have become increasingly popular to use, alone or in combination with other molecules. This review offers an overview of the physicochemical and biological properties of chitosan and its derivatives. These are useful for different applications, focusing on the delivery of therapeutic molecules and regenerative approaches in the CNS. A literature review was performed through online platforms as PubMed, ScienceDirect, and the National Library of Medicine (clinicaltrials.gov), considering only the works published in the last 5 years. This review aims to show the reader the current trends and limitations of this biopolymer in biomedical applications directed toward the CNS.

## Chitosan

Chitosan is a polysaccharide mainly composed of D-glucosamine and, in a lower proportion, N-acetyl-D-glucosamine units randomly β-(1-4)-linked. It can be obtained by deacetylation processes of chitin, which has been recognized as the second most abundant polysaccharide in nature, after cellulose. Even though the main source of chitin is crustacean shell, recent technologies have made possible the obtention of chitin and chitosan from other sources like insects and microorganisms (Peniche et al., 2008; Zargar et al., 2015). Particularly, fungal sources have gained increased attention due to some potential advantages like a homogeneous polymer length, a high degree of deacetylation, and high solubility (Ghormade et al., 2017). In general, there are two types of processes to obtain chitosan: chemical and biological. The chemical method is the most commonly performed at an industrial scale, using strong acid and alkaline treatments (El-Knidri et al., 2018). Biological methods involve microorganisms and enzymes (Arbia et al., 2013), but despite the efforts to achieve scalable enzymatic deacetylation, the high crystallinity of chitin remains the main obstacle (Jaworska and Roberts, 2016).

The source and obtention process of chitosan are important factors to consider according to the desired application. These factors define the final product characteristics. For biomedical applications, its purity, molecular weight (Mw), crystallinity, and deacetylation degree (DD) are of great importance (Nwe et al., 2009). These factors deeply correlate with chitosan’s mechanical and biological properties. Aranaz et al. (2009) reported the relationship between the physicochemical properties of chitosan and its behavior in biomedical applications. This will be further detailed for each biomedical application described in this work.

The increasing interest in chitosan as a biomaterial is due to its natural origin and several biological properties: biocompatibility, non-toxicity, non-allergenicity, and biodegradability, as well as its antifungal, antibacterial, antioxidant, anti-tumor and anti-inflammatory activities. Besides, it has been recognized as an immunoadjuvant, anti-thrombogenic and anti-cholesteremic agent (Younes and Rinaudo, 2015; Kim, 2018). It also possesses high versatility, so it can be used in many physical forms as fibers (and nano-fibers), gels, sponges, beads, films, particles (and nanoparticles), membranes and scaffolds (El-hefian et al., 2011; Rebelo et al., 2017). All these properties make chitosan adequate for many biomedical applications as drug delivery, gene delivery, tissue engineering, and regenerative therapies, among others. However, when it is used on its own, it has poor mechanical properties in wet conditions and low solubility at pH > 7.0. This situation has led to the search of different strategies to overcome chitosan deficiencies by its combination with other materials or through changes in its superficial structure.

It is important to highlight that chitosan is a polycationic polymer, this attribute is conferred by the protonation of D-glucosamine which forms a positively charged moiety (NH_3_^+^) at neutral/physiological pH (Muanprasat and Chatsudthipong, 2017). Despite the versatility that this characteristic gives to chitosan, cationic polymers have been reported as neurotoxic and CNS damage inducers (Li and Ju, 2017). The neurotoxicity has been associated with chitosan’s particle size through inflammasome activation (Bueter et al., 2011). However, the evaluation of chitosan’s neurotoxicity is still limited and not detailed. On the other hand, chitosan and its derivatives have also been reported as neuroprotective over different neuronal disorders including Alzheimer’s and Parkinson’s disease, sclerosis, stroke, and injury, among others (Pangestuti and Kim, 2010; Hao C. et al., 2017; Ouyang et al., 2017).

## Chitosan Derivatives

**FIGURE 1:**
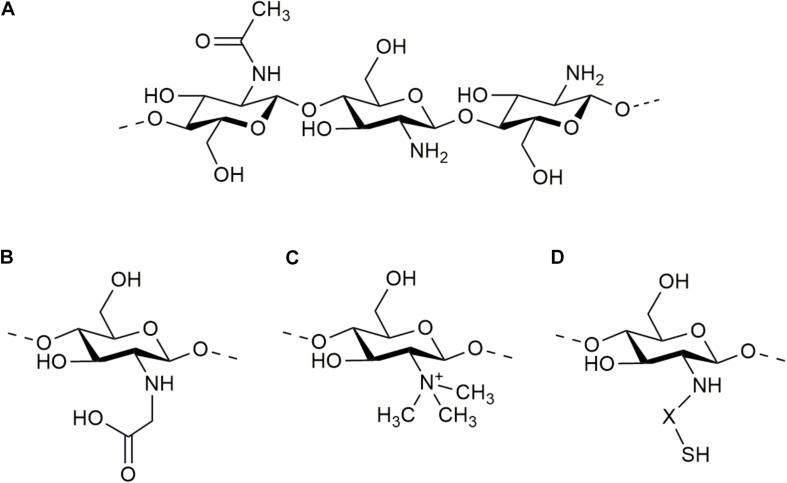
Molecular structure of chitosan **(A)** and some of its derivatives: N-carboxymethyl chitosan **(B)**, N-trimethyl chitosan **(C)**, and thiolated chitosan **(D)**.

The molecular structure of chitosan’s units contains an amino/acetamido group at C-2, a secondary hydroxyl group at C-3 and a primary hydroxyl group at C-6 (Figure 1A). So, the improving modifications that have been developed for this polymer make use of these groups by grafting other molecules. Some of these modifications consist of carboxyalkylation, thiolation, sulfation, phosphorylation, esterification, graft copolymerization, and cross-linking strategies (Mourya and Inamdar, 2008; Muñoz and Zuluaga, 2017). These modifications confer new and unique properties to the obtained products. For example, chitosan has been grafted with heparin to increase its anticoagulant and angiogenic properties, and to increase its affinity for growth factors (Skop et al., 2019). It has also been grafted with laminin-derived peptides to facilitate the attachment of neurons and neurite outgrowth (Kuo and Chiu, 2011). Many molecules can be grafted to improve the application of chitosan to the CNS but have not been evaluated yet. Dicarboxylic acids contain two binding sites that can lead to the crosslinking of chitosan polymeric chains and offer an antioxidant environment. In the same way, hydroxycinnamic acids possesses an important antioxidant activity. For example, the release of ferulic acid into the lesion site of traumatic brain injury (TBI) has shown to effectively protect further secondary injury through the inhibition of neurological oxidative stress (Dong et al., 2015). Nevertheless, these acids have mainly been grafted to chitosan to modify its physicochemical characteristics as solubility, thermal stability, or rheological properties, and have not been widely studied into the CNS (Liu et al., 2017).

Among the most commonly used modifications of chitosan for biomedical applications directed to CNS are carboxymethylation, N-trimethylation, and thiolation (Figures 1B–D, respectively). These modifications confer new properties to chitosan, as solubility and mucoadhesiveness, converting these biomaterials into proper substrates for biomolecule delivery. Chitosan graft copolymerization is also widely used for CNS application, because it allows to obtain polymers with controlled structures and activities. These are defined by the graft characteristics, including the structure of the molecule, its length, and number (or binding degree). Copolymerization is widely used for the elaboration of tailor-made scaffolds (Mourya and Inamdar, 2008). In addition to the graft attributes, the binding site also plays an important role in the final properties of chitosan-based biomaterials. Ding et al. (2014) produced 6-O-sulfated chitosan and observed a strong effect of the sulfate site in promoting the neural differentiation of mouse embryonic stem cells.

### Carboxymethyl Chitosan (CMC)

The introduction of carboxyalkyl groups into the structure of chitosan, as carboxymethyl, has been developed mainly as a strategy for increasing chitosan’s solubility. The reaction occurs either at the C6 hydroxyl group or at the NH_2_ moiety, giving N-CMC, O-CMC or N,O-CMC as products. These derivatives are amphoteric polymers that produce water-soluble compounds with pH-dependent solubility, water retention properties, biodegradability, biocompatibility, and antioxidant activity (Muñoz and Zuluaga, 2017; Shariatinia, 2018; Xu et al., 2018). Therefore, these amphoteric polymers can be loaded with hydrophobic drugs and display strong bioactivity (Upadhyaya et al., 2014). Moreover, the presence of the functional –OH, –NH_2_, and –COOH groups in its structure gives the possibility of being easily modified. For example, CMC has been crosslinked with alginate and agarose to be used as a scaffold for stem cell *in situ* differentiation into functional neurons and supporting neuroglia (Gu et al., 2016). CMC has also been employed to enhance the efficacy of active constituents with poor solubility and bioavailability, and increase brain drug concentration (Ding et al., 2016; Liu et al., 2018). However, Wahba and collaborators developed a galantamine delivery system, against Alzheimer’s disease, attaching galantamine to ceria-containing hydroxyapatite as well as ceria-containing CMC-coated hydroxyapatite nanocomposites. They found that the CMC coating delayed the *in vitro* release for galantamine and nanoceria (Wahba et al., 2016).

### N-Trimethyl Chitosan (TMC)

Methylation consists in the introduction of various alkyl groups at the amino groups of chitosan. The most common product of these reactions is TMC, which is considered one of the strongest mucoadhesive polymers due to its cationic nature (M Ways et al., 2018). That is why it has been used for brain-targeting drug delivery, showing great potential in nose-to-brain applications (Kumar et al., 2013; Meng et al., 2018; Pardeshi and Belgamwar, 2018). Another promising application of TMC is its use to treat brain tumors. For example, Turabee and his team found that the addition of TMC to a pluronic F127 hydrogel increased the biological activity of docetaxel against U87-MG cells. The pluronic F127-TMC/docetaxel hydrogel was evaluated *in vivo* employing BALB/c nude mice and showed sustained release of docetaxel with tumor suppression (Turabee et al., 2019). Similarly, Sedeky et al. (2018) observed a significant improvement in cytotoxicity of Piperine-loaded TMC nanoparticles on human brain cancer cell line Hs683.

### Thiolated Chitosan

Thiolation is the reaction of primary amino groups of chitosan with coupling reagents that contain thiol groups (thioglycolic acid, 2-iminothiolane, cysteine, and thiobutylamidine). This product has high permeation, mucoadhesion, higher solubility at physiological pH and displays *in situ* gelling properties (Sreenivas and Pai, 2008). These properties present thiolated chitosan as a good substrate for drug delivery to the brain, mainly used as nanoparticles (Patel et al., 2012, 2013; Singh et al., 2016; Sunena et al., 2019). In this way, Patel et al. (2013) studied brain uptake of cyclobenzaprine HCl-loaded thiolated chitosan nanoparticles on Swiss albino mice after intranasal administration and observed that thiolation of chitosan reduced trans-mucosal toxicity and enhanced the bioavailability. The *in situ* gelling ability makes thiolated chitosan suitable not only for nose-to-brain applications but also for the elaboration of scaffolds. However, it has not been widely used for neural tissue engineering. For this purpose, methacrylamide chitosan has been thiolated, giving as products porous and biodegradable scaffolds that are suitable for cell growth and neural stem cell differentiation in 3D (Yu et al., 2007; Leipzig et al., 2011).

### Grafting Copolymerization of Chitosan

Frequently, chitosan is grafted with other polymers to reach copolymerization. The graft polymer is selected by its chemical, mechanical or biological properties and the copolymerization results in a chitosan-based product with added characteristics. For example, polyethylene glycol (PEG)-grafted chitosan derivatives have increased solubility over a wide range of pH and have shown enhanced mucoadhesion (Bhavsar et al., 2017). In this way, 2-O-PEGylated chitosan has been used for the elaboration of siRNA-carrying nanoparticles that target the brain to treat neurodegenerative diseases (Malhotra et al., 2013a). Other polymers that have been grafted to chitosan for CNS application are gelatin (Gao S. et al., 2014), poly lactic-co-glycolic acid (PLGA) (Tong et al., 2017), poly (3,4 ethylenedioxythiophene) (PEDOT) (Wang S. et al., 2018), alginate, and agarose (Gu et al., 2016), among others.

## Chitosan-Based Delivery Systems to CNS

**FIGURE 2:**
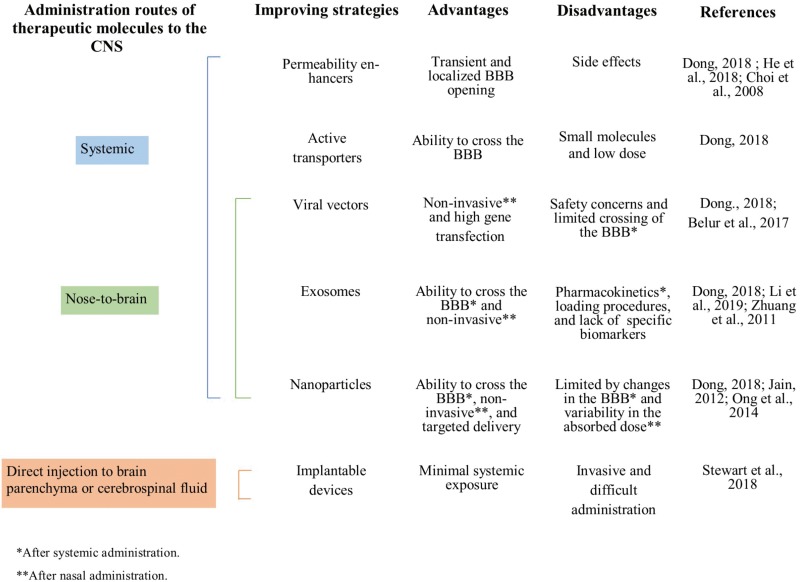
Advantages and disadvantages of the current strategies to enhance therapeutic molecules delivery to the CNS.

For many years, the increasing incidence of neurodegenerative disorders and the lack of functional treatments have encouraged the search for new therapeutic approaches to counteract CNS diseases. The administration routes directed to the CNS mainly consist of systemic administration, nose-to-brain, and direct injection into the brain parenchyma or cerebrospinal fluid. However, it remains challenging to find effective treatments. One of the main reasons for this is the BBB, which separates the brain from the blood supply and distinguishes between the molecules that can and cannot cross through itself. The BBB allows the entry of nutrients and hormones but restricts other external materials. Therefore, most of the therapeutic molecules are unable to cross and access to the CNS from the bloodstream, following systemic administration (Chatterjee et al., 2019). This situation has led to the development of different strategies for aiding therapeutic molecules to permeate the BBB and to get access to the brain. Dong elaborated a review article providing an overview of the current strategies to enhance drug delivery to the brain (Dong, 2018). According to it, permeability enhancers, active transporters, viral vectors, nanoparticles, and exosomes have been proposed for aiding therapeutic molecules to cross the BBB after systemic administration (Choi et al., 2008; Dong, 2018; He et al., 2018). For direct administration into the brain parenchyma or cerebrospinal fluid, implantable devices have emerged as effective delivery systems that avoid systemic concerns (Stewart et al., 2018). However, most of these strategies have the disadvantage of bearing low drug concentrations or being invasive. Therefore, the use of carriers/vehicles and non-conventional administration routes have emerged as a new approach for facilitating the delivery of therapeutic molecules to the brain (Upadhyay, 2014; Bonferoni et al., 2019). In this way, nose-to-brain administration has also made use of viral vectors, exosomes, and nanoparticles to achieve less invasive and more effective treatments (Jain, 2012; Belur et al., 2017; Dong, 2018; Li et al., 2019). This information is summarized in Figure 2. Nevertheless, the use of biocompatible carriers is encouraged to prevent unwanted effects and achieve high and sustained local drug delivery (Chen et al., 2019).

### Drug Delivery to CNS

Chitosan possesses a lot of advantages as a brain-targeted drug carrier. Coupled with its capability to penetrate the BBB, it also can control release, adhere to mucus, and open tight junctions of the nasal membrane. These abilities favor its application in nose-to-brain drug delivery strategies (Mohammed et al., 2017; Yu et al., 2019).

Another advantage of chitosan is its versatility, it can be used for drug delivery purposes as microspheres, capsules, hydrogels, conjugates, nanoparticles, films, beads, or tablets (Ali and Ahmed, 2018). However, nanoparticles have gained special attention in this field due to their capability to protect drugs from degradation during administration (Tzeyung et al., 2019). Chitosan nanoparticles have shown to enhance the brain targeting efficiency and, therefore, to improve the therapeutic potential of drugs (Md et al., 2013; Nagpal et al., 2013). Chitosan has also been used as a nanoparticle coating, to grant drug-loaded nanoparticles with a net positive charge and facilitate cellular internalization (Varan and Bilensoy, 2017).

For drug delivery, it has been reported that the use of low Mw chitosan increases the encapsulation efficiency (Yang and Hon, 2009), reduces cytotoxicity and increases the degradation rate of nanoparticles, properties that have been also associated with higher DDs (Sarvaiya and Agrawal, 2015). On the other hand, the penetration in the mucin layer and the mucoadhesion strength of chitosan increase when the Mw is higher (Rassu et al., 2016). It is worth mentioning that these properties are influenced when chitosan is functionalized, and they depend on the added molecules. For example, Kuo and collaborators, recently developed chitosan-PLGA nanoparticles grafted with anti-aldehyde dehydrogenase and sialic acid for brain tumor-targeted delivery of curcumin (Kuo et al., 2019). They promoted the BBB permeation through N-acetylglucosamine. However, the targeting of the delivery system was improved with the addition of sialic acid and the anti-aldehyde dehydrogenase by directing it to the membrane of glioblastoma cells and brain cancer stem cells.

The use of chitosan and its derivatives for drug delivery to the brain has been employed for developing treatments against many neurological disorders, mainly for Parkinson’s and Alzheimer’s diseases. Other studies have been guided to treat conditions like depression, schizophrenia, migraine, brain tumor, general anxiety disorder, epilepsy, pain, viral and bacterial infections, and so on (Table 1). However, at the time of writing this article, only one of these studies has been taken to clinical trials (Ruppen, 2015). In that clinical research, a nasal ketamine spray with chitosan was evaluated in comparison with oral morphine to treat pain in cancer outpatients but no results have been reported yet.

**TABLE 1:** Chitosan drug delivery systems for brain targeting reported in the last 5 years.

Drug	Presentation	Application	Administration route	References
Pentamidine	Chitosan coated niosomes	Parkinson’s disease	Intranasal	Rinaldi et al., 2019
Methotrexate	Chitosan hydrogel nanoparticles	Antineoplastic agent	Intravenous	Pourtalebi-Jahromi et al., 2019
Carbamazepine	Chitosan coated lipid nanoparticle formulation	Epilepsy	Oral	Ana et al., 2019
Rotigotine	Chitosan nanoparticles	Parkinson’s disease	Intranasal	Tzeyung et al., 2019
Doxorubicin/erlotinib	Chitosan liposomal nanoparticles	Glioblastoma	–	Lakkadwala and Singh, 2019
Docetaxel	TMC hydrogel	Glioblastoma	Intracranial injection	Turabee et al., 2019
Risperidone	Chitosan lipid nanoparticle	Schizophrenia	Intranasal	Qureshi et al., 2019
Pramipexole dihydrochloride	Chitosan nanoparticles	Parkinson’s Disease	Intranasal	Raj et al., 2018
Galantamine	Chitosan nanoparticles	Amnesia/Alzheimer	Intranasal	Sunena et al., 2019
Selegiline	Chitosan nanoparticles	Parkinson Disease	Intranasal	Sridhar et al., 2018
Temozolomide	Nano lipid chitosan hydrogel	Antineoplastic agent	Intranasal	Khan et al., 2018
Cyclovirobuxine d	Chitosan nanoparticles	Cerebrovascular disease	Intranasal	Wei et al., 2018
Diazepam	Chitosan mucoadhesive microemulsion	Status epilepticus	Intranasal	Ramreddy and Janapareddi, 2019
Tapentadol hydrochloride	Chitosan nanoparticles	Pain	Intranasal	Javia and Thakkar, 2017
Rivastigmine hydrogen tartrate	Chitosan mucoadhesive microemulsion	Cholinesterase inhibitor	Intranasal	Shah et al., 2018
Ribavirin	Chitosan microparticle agglomerates	Viral infection	Intranasal	Giuliani et al., 2018
Huperzine A	Lactoferrin-conjugated TMC surface-modified PLGA nanoparticles	Alzheimer’s disease	Intranasal	Meng et al., 2018
Ropinirolle-detran sulfate	Chitosan mucoadhesive neuro-nanoemulsion	Parkinson’s disease	Intranasal	Pardeshi and Belgamwar, 2018
Zolmitriptan	Chitosan mucoadhesive nanoemulsion	Migraine	Intranasal	Abdou et al., 2017
Desvenlafaxine	PLGA-chitosan nanoparticles	Depression	Intranasal	Tong et al., 2017
Selegiline hydrochloride	Thiolated chitosan nanoparticles	Depression	Intranasal	Singh et al., 2016
Quetiapine fumarate	Chitosan microemulsion	Schizophrenia	Intranasal	Shah et al., 2016
Rasagiline	Chitosan glutamate nanoparticles	Parkinson’s disease	Intranasal	Mittal et al., 2016
Ropinirole hydrochloride	Chitosan mucoadhesive nanoparticles	Parkinson’s disease	Intranasal	Jafarieh et al., 2015
Buspirone hydrochloride	Thiolated chitosan nanoparticles	General anxiety disorder	Intranasal	Bari et al., 2015
Doxepin hydrochloride	Chitosan-glycerophosphate-PEG thermoreversible biogels	Depression	Intranasal	Naik and Nair, 2014
Buspirone	Chitosan mucoadhesive microemulsion	General anxiety disorder	Intranasal	Bshara et al., 2014
Donepezil	Chitosan nanosuspension	Alzheimer disease	Intranasal	Bhavna et al., 2014
Levodopa	Chitosan nanoparticles	Parkinson’s Disease	Intranasal	Sharma et al., 2014

### Gene Therapy

Gene therapy has been set as a form of drug delivery, where cellular machinery is modulated to produce a therapeutic effect (Blau and Springer, 1995). As in drug delivery systems, some of the most remarkable difficulties to direct this technology toward the CNS consist of low BBB permeability, brain heterogeneity, invasive or inefficient routes of administration, and dosing (Joshi et al., 2017). Different types of vectors have been used to overcome these limitations, being the viral ones the most employed (Choudhury et al., 2017). Nevertheless, human infections and immune response caused by viral vectors have led to the search for safer vectors. The aforementioned polycationic property of chitosan confers the polymer the capacity to establish strong electrostatic interactions with negatively charged molecules, like DNA and RNA.

To this day many chitosan-based systems for gene delivery have been employed. Mao et al. (2010) reviewed the formulation factors that affect siRNA and DNA delivery and transfection efficiency. They highlighted that the transfection efficiency depends on many parameters and concluded that intermediate values of Mw and DD of chitosan form complexes of intermediate stability and efficient transfection. Chitosan derivatives have also been employed for this purpose. Specifically, for therapeutic gene delivery to the brain, PEGylation has shown to enhance biocompatibility and stability of siRNA loaded complexes (Gao Y. et al., 2014). Moreover, PEG plays the role of a linker between chitosan and targeting peptides, which form complexes with nucleic acid and enhance the cellular uptake of chitosan nanoparticles (Malhotra et al., 2013b; Jiang et al., 2014).

Despite the versatility of chitosan, nanoparticles have been the preferred candidates to counter different neurological disorders. Among these disorders are glioblastoma (Malmo et al., 2013; Danhier et al., 2015; Xu et al., 2015; Van Woensel et al., 2016, 2017), medulloblastoma (Kievit et al., 2015), Parkinson’s disease (Peng et al., 2014), Alzheimer’s disease (Gao Y. et al., 2014; Rassu et al., 2017), and multiple sclerosis (Youssef et al., 2019). Even viral infections, like HIV-infected brain, have been a target for this therapeutic strategy (Gu et al., 2017). Recently, the search for less invasive strategies has guided the development of novel formulations for nose-to-brain gene delivery. For example, Rassu et al. (2017), made chitosan-coated solid lipid nanoparticles carrying BACE1 siRNA for intranasal application against Alzheimer’s disease. Similarly, Van Woensel et al. (2017) formulated siRNA targeting Gal-1 loaded chitosan nanoparticles for intranasal delivery in mice, obtaining remarkable changes in the tumor micro-environment of glioblastoma multiforme. Moreover, Sánchez-Ramos and his collaborators designed a chitosan-Mangafodipir intranasal nanocarrier system for the delivery of siRNA or dsDNA. They employed anti-eGFP siRNA and reported the effectiveness of the nanoparticles for reducing GFP mRNA expression in Tg GFP+ mice along different brain zones (Sanchez-Ramos et al., 2018). These advances suggest an imminent overcoming of the difficulties that limit the CNS-directed gene therapy.

## Chitosan-Based Materials for Tissue Engineering and Regenerative Medicine in CNS

The design of different chitosan-based biomaterials for tissue engineering and regenerative medicine in CNS aims to facilitate neural cell adhesion, proliferation, and differentiation. These biomaterials can be used as scaffolds to mimic the natural extracellular matrix and microenvironment for better *in vitro* approaches or tissue replacement. Thus, these polymeric materials can be useful to overcome the limitations of cell therapy. For *in vitro* applications, the conformation of the biomaterials must present good biocompatibility and porous structures that favor 3D cell growth. Regenerative medicine requires biomaterials that offer mechanical support for growing neurites. Biological support is also required to lead the processes to tissue restoration through stem cell differentiation and integration into the surrounding healthy tissue (Boni et al., 2018). Moreover, it is important to cause a minimal inflammatory response when implanted. In this way, properties as biocompatibility, biodegradability, mechanical strength, architecture, and cell-adhesion capacity become crucial for biomaterial success.

Gnavi et al. (2013) made a review article detailing the characteristics of chitosan-based scaffolds for nervous system regeneration. They highlighted that the physicochemical properties of chitosan (and modified chitosan) can be easily manipulated to design specific structural features for the scaffolds. According to the required structure and properties for tissue restoration, the biomaterial scaffolds for CNS regeneration can be classified into two types: hydrogels and biodegradable scaffolds (Wang Y. et al., 2018). Chitosan hydrogels can be obtained by physical or chemical crosslinking. The physical associations, like ionic bonding and hydrogen bonds, provide unstable structures while the chemical associations formed by covalent bonds give place to uniform properties. For faster hydrogel biodegradation, it is recommended the use of labile bonds that can be broken under physiological conditions (Pellá et al., 2018). On the other hand, *in situ* gelling can be achieved by physical interactions, providing the advantages of cell delivery without previous geometrical shape preparation of hydrogels and with a less invasive implantation process (Shariatinia and Jalali, 2018). For application in the CNS, hydrogels have been obtained from chitosan (Chedly et al., 2017), its derivatives as CMC (Xu et al., 2018) and chitosan lactate (Nawrotek et al., 2017), and mixtures with other polymers like gelatin (Gao S. et al., 2014). Biodegradable scaffolds are mainly structured by freeze-drying but can be also obtained by electrospinning, solvent evaporation, supercritical carbon dioxide, and 3D printing (Croisier and Jérôme, 2013; Wang Y. et al., 2018; Sun et al., 2019). For porous scaffolds, many chitosan-blends have been made by combining different biodegradable materials, like gelatin (Wang et al., 2017), collagen (Yan et al., 2019), and PEDOT (Wang S. et al., 2018), among others. One of the principal advantages of these scaffolds resides in having stabilized porous structures that can be designed with different size ranges and mechanical properties (Xu et al., 2017).

For non-CNS tissue engineering applications, Mw and DD have been associated with biodegradability and viscosity. Higher Mw gives delayed biodegradation when implanted, and more viscous biomaterials. DD values between 65 and 82% give faster biodegradation (Rodriguez-Vazquez et al., 2015). It is worth mentioning that there is huge variability in the main chemical properties of the starting chitosans used in the reviewed studies, including Mw from 1 (Yao et al., 2018) to 550 kDa (Chedly et al., 2017) and values of 75–95% of DD (Feng et al., 2014; Tseng et al., 2015). Moreover, none of the reviewed CNS application studies in tissue engineering and regenerative medicine evaluates different Mw or DD in their starting materials. Many of the studies do not detail these two important chemical characteristics of their starting chitosan. Otherwise, the main variation in these works consists of using different polymeric blends and ratios as starting materials.

Implanting chitosan-based biomaterials in the CNS provides a way to its poor regenerative capacity through the reconstruction of lost tissue and reconnection of neuronal processes. Although, the incorporation of stem cells and biomolecules into these scaffolds has emerged as an additional strategy to enhance regenerative therapies (Ricks et al., 2014). In this way, biomaterials assist cell therapy as delivery vehicles that promote cell survival and engraftment. Another advantage of the combination of both research areas is that the implanted cells can be separated from the host damaged tissue. Thereby, biomaterials provide an independent microenvironment for cell differentiation and proliferation, which does not occur in the natural response to damage (Wang Y. et al., 2018).

Beyond the aforementioned physicochemical properties of chitosan that make it a suitable biopolymer to make biodegradable scaffolds and hydrogels, chitosan has neuroprotective properties. Anti-neuroinflammatory activity, suppression of β-amyloid and acetylcholinesterase formation, and anti-apoptosis effects have been reported (Pangestuti and Kim, 2010; Hao C. et al., 2017). These neuroprotective effects promote an adequate microenvironment for cell proliferation in some CNS damage processes.

### Chitosan-Based Scaffolding

Over the past few years, many strategies for increasing cell adhesion, differentiation and viability on chitosan-based scaffolds have been implemented (Table 2). Different mixtures of biopolymers with chitosan have been employed for modulating the micro-structure of the scaffolds and their properties. For example, collagen copolymerization has proven to promote cell affinity through its arginine-glycine-aspartic acid sequence which is recognized by transmembrane integrins (Kuo and Yeh, 2011). In the same way, polylactic acid copolymerization gives rise to materials with better mechanical properties and it has been cataloged as a perfect synthetic polymer to elaborate composite materials with chitosan (Ebrahimi-Barough et al., 2015; Hoveizi et al., 2015). On the other hand, Abasi et al. (2019) recently developed bionanocomposites of polyaniline-chloride/chitosan and observed that physical factors of the scaffolds (as electrical conductivity and morphology) have a bigger influence in cell-substrate interactions than molecular affinity. Also, Sung et al. (2015) studied the behavior of Neuro-2a cells over flat, micro-, and nano-textured chitosan substrates, and found that cellular adhesion increases over flat chitosan surfaces. Given that the design of the internal structure and surface of the scaffolds is determinant for cell adhesion and proliferation, Sun and collaborators printed a collagen-chitosan 3D scaffold with a specific structure. They observed nerve fibers regeneration and functional recovery after its implantation in rats with spinal cord injury (SCI), showing enhanced therapeutic effects compared with the non-3D-printed material (Sun et al., 2019).

**TABLE 2:** Chitosan-based biomaterials for implantation in CNS or neural cell culture reported in the last 5 years.

Composition	Presentation	Application	Model	References
Collagen and chitosan	3D printed scaffolds	Implantation as therapeutic in SCI	Rat	Sun et al., 2019
Chitosan-multiwalled carbon nanotubes	Nanomaterial scaffold	Culture for implantation	*In vitro*	Gupta et al., 2019
Polyaniline-chloride, chitosan, and NGF	Microporous scaffolds	Tissue engineering	*In vitro*	Abasi et al., 2019
Gelatin and glycine-functionalized polypyrrole-coated poly(vinyl alcohol) with chitosan	Scaffold	Culture for implantation	Mice	Naghavi-Alhosseini et al., 2019
PEDOT, chitosan and gelatin	Scaffold	Substrate for NSC research and neural tissue engineering	*In vitro*	Wang S. et al., 2018
Chitosan and PDGF	Scaffold and microspheres	Tissue-engineered spinal cord grafts	*In vitro*	Chen et al., 2018
Chitosan	Scaffold	Implantation in SCI	Rat	Yao et al., 2018
NT-3 – chitosan	Tube	Implantation in SCI	Monkey	Rao et al., 2018
PEDOT and CMC	Conductive polymer layer/Hydrogel	Neural tissue engineering	*In vitro*	Xu et al., 2018
Chitosan and heparin	Scaffold	Culture of stem cells for implantation	*In vitro*	Moore et al., 2018
NT-3 – chitosan	Chitosan particles	Implantation in TBI	Rat	Hao P. et al., 2017
Chitosan	Fragmented physical hydrogel suspensión	Implantation in SCI	Rat	Chedly et al., 2017
Chitosan lactate	Hydrogel	Implantation in SCI	Rat	Nawrotek et al., 2017
Polyacrylamide, chitosan scaffold, and PLGA nanoparticles	Inverted colloidal crystal scaffold	Culture for iPS differentiation into neurons and implantation for nerve regeneration	*In vitro*	Kuo and Chen, 2017
Alginate, CMC, and agarose	Porous 3D scaffold	Tissue engineering	*In vitro*	Gu et al., 2016
Chitosan and polylactic acid	Nanofibrous scaffold	Culture of stem cells for tissue engineering and cell-based therapy	*In vitro*	Ebrahimi-Barough et al., 2015
NT-3 – chitosan	Tube	Implantation in SCI	Rat	Duan et al., 2015
Chitosan	Scaffold	Culture of stem cells for differentiation and implantation in TBI	*In vitro*	Feng et al., 2014

The addition of neurotrophic factors into chitosan scaffolds or microspheres, like nerve growth factor (NGF), neurotrophin-3 (NT-3), or fibroblast growth factor-2 (FGF-2), has shown to enhance neurogenesis, neural differentiation, and cell survival (Yi et al., 2011; Skop et al., 2013; Duan et al., 2015; Hao P. et al., 2017). Rao et al. (2018) elaborated NT-3 – chitosan tubes that promoted neuroprotection, neurogenesis, revascularization, and antiinflammation on SCI conditions. After implantation, they observed robust neural regeneration with motor and sensory functional recovery in rhesus monkeys (Rao et al., 2018).

The implantation of chitosan hydrogels constitutes an interesting possibility for CNS restoration. Chitosan hydrogels have proved to provide a suitable micro-environment for axons regrowth and increase the survival rate of damaged neurons in different animal models. These hydrogels have shown remarkable potential in CNS repair, even in the absence of added trophic factors or without a detailed design of its structure (Tseng et al., 2015; Nawrotek et al., 2017). Chedly et al. (2017) elaborated a fragmented physical hydrogel suspension employing unmodified chitosan for its implantation in rat SCI (immediately after the injury). They observed axonal regrowth, modulated inflammatory response, and long-lasting locomotor function recovery (Chedly et al., 2017). Even though more studies employing chitosan hydrogels are required to define their therapeutic potential in different damage models or degenerative diseases, these results provide a tool for future evaluations in combined repair strategies.

### Chitosan-Based Materials and Cell Therapy to the CNS

Spinal cord and brain injury, as well as neurodegenerative diseases, are conducted by different biological processes and cause diverse symptoms, though all of them result in neuronal degeneration and cell death. Cell therapies for CNS have attained clinical research in different pathological conditions like stroke, TBI, amyotrophic lateral sclerosis, and Parkinson’s disease, showing their contribution to mitigating damage (Watanabe, 2018). However, within damage processes occur extracellular matrix, neuronal, and glial cell loss. This tissue loss results in a hostile environment for transplanted cells and causes deficient engraftment with poor cell viability (Boisserand et al., 2016). In recent years, the incorporation of different biomaterials to cell therapy in CNS has shown to promote cell survival, integration, and differentiation (Führmann and Shoichet, 2018). In this way, chitosan-based biomaterials have been employed in combination with stem/precursor cells to build a way to neuro-regeneration (Table 3). The function of these biopolymeric structures is not only to serve as delivery vehicles and cell physical supports, besides they must regulate the biological microenvironment to guide axonal growth and favor the integration of the healthy tissue to the lesion zone (Boni et al., 2018). Some of the most studied cells for CNS repair are the mesenchymal stem cells (MSC), bone marrow mesenchymal stem cells (BM-MSC), neural stem cells (NSC), and neural precursor cells (NPC).

**TABLE 3:** Chitosan-based biomaterials for CNS cell therapy reported in the last 5 years.

Composition	Cells	Presentation	Application	Model	References
Collagen and chitosan	BM-MSC	Porous scaffold	Implantation in TBI	Rat	Yan et al., 2019
Polyaniline-chloride, chitosan, and NGF	PC12/NIH/3T3	Microporous scaffold	Neural tissue engineering	*In vitro*	Abasi et al., 2019
Chitosan, genipin, heparin, FGF-2, and fibronectin	NPC/genetically modified NPC	Microspheres	Implantation as therapeutic in TBI	Rat	Skop et al., 2019
Poly(ε-caprolactone), chitosan, and polypyrrole	PC12	Nanofibrous scaffold	Neural tissue substitute	*In vitro*	Sadeghi et al., 2019
Chitosan	BM-MSC	Porous scaffold	Implantation in TBI	Rat	Tan et al., 2018
Methacrylamide chitosan, dibenzocyclooctyne-acrylic acid, and laminin azide-tagged interferon γ	NSC	Conduit	Implantation in SCI	Rat	Farrag and Leipzig, 2018
PEDOT, gelatin, and chitosan	NSC	Scaffold	Neural tissue engineering	*In vitro*	Wang et al., 2017
Chitosan	NSC and MSC	Co-spheroids	Implantation in TBI	Zebrafish	Han and Hsu, 2017
Chitosan	MSC from dental pulp	Scaffold	Implantation in SCI	*In vitro*	Zhang et al., 2016
Chitosan	MSC	Scaffold	Implantation in SCI	Rat	Kim et al., 2016
Chitosan, genipin, heparin, fibronectin, and FGF-2	Retinal ganglion cells	Microspheres	Cellular and growth factor delivery vehicle in TBI	Rat	Skop et al., 2016
Chitosan and gelatin	BM-MSC	Scaffold	Implantation in spina bifida	Rat fetuses	Li et al., 2016
Chitosan and collagen	BM-MSC	Scaffold	Implantation in ischemic stroke	Rat	Yan et al., 2015
Chitosan, polylactic acid, NGF, and bGFG	PC12	Scaffold	Neural cell differentiation for transplantation in a MS model	Mice	Hoveizi et al., 2015
Glycol chitosan and DF-PEG	NSC	Self-healing hydrogel	Implantation in neural injury	Zebrafish embryo	Tseng et al., 2015
Methacrylamide chitosan, collagen, IFN-y, and acrylated laminin	NSC	Conduit	Implantation in SCI	Rat	Li et al., 2014
Chitosan and gelatin	MSC from human adipose tissue	Scaffold	Implantation in TBI	Mice	Gao S. et al., 2014

A study reported by Sugai et al. (2015) showed that modified-chitosan microfibers promote neural stem/progenitor cell proliferation *in vitro* but not cell survival after transplantation, contrary to collagen-based microfibers. The authors proposed that the stiffness of chitosan precluded the colonization of other cells, like vascular epithelial cells (Sugai et al., 2015). The stiffness of the scaffolds should be in the range of 0.1–1 kPa for mimicking soft tissue like the brain. It is well known that stiffness has a notorious influence on stem cell response and function (Liang et al., 2019). Moreover, it has been proved that cell size affects the cellular response to matrix stiffness in 3D cultures, especially large cells as many of the human stem cells (Bao et al., 2019). So, in these cell-scaffold strategies, it is very important to consider the starting material and cell population. The main advantage of using chitosan as the starting material for this purpose is its high versatility. Thus, stiffness and other important characteristics that affect cell behavior, as viscoelasticity, porosity, and topography can be easily modulated.

Besides its versatility, chitosan and its derivatives have shown to be a superior substrate for cell therapy in comparison with other polymers. Scanga et al. (2010) showed the capability of adult murine NPC to proliferate and differentiate into the three neural cell types when they were cultured over chitosan hydrogel films. On the contrary, NPC differentiation was not observed over poly(oligoethylene oxide dimethacrylate-*co*-2-amino ethyl methacrylate) or its blend with poly(vinyl alcohol), neither over poly(glycerol dimethacrylate-*co*-2-amino ethyl methacrylate) (Scanga et al., 2010). Kim et al. (2016) observed a better functional improvement in rats with SCI after MSC transplantation over chitosan scaffolds in contrast to PLGA scaffolds. Moreover, they studied intralesional injection of the same cells and compared it with scaffold-based transplantation in rats. They found a higher MSC engraftment when the scaffolds were employed (Kim et al., 2016). The culture of rat PC12 line and human neural stem cells over chitosan has also shown better results in comparison with cellulose acetate or polyethersulfone derived electrospun nanofibers (Du et al., 2014).

The incorporation of trophic factors as FGF-2, NGF, PDGF, and bGFG has also shown to enhance stemness of neural stem cells and favor its differentiation and proliferation when implanted with chitosan scaffolds or microspheres (Hoveizi et al., 2015; Skop et al., 2016; Moore et al., 2018). Recently, Skop and his collaborators designed a cell-scaffold strategy employing a radial glial neural precursor cell line that conditionally secreted insulin-like growth factor I. This cell line was attached to a chitosan-based microsphere scaffold and injected into the lesion cavity of adult rats with TBI. They observed differentiation toward the three neural cell types (neurons, astrocytes, and oligodendrocytes) and improved capacity for neuronal differentiation. These obtained effects led to the recovery of the somatosensory function. However, the presence of insulin-like growth factor I was not associated with a higher cell retention rate or improved cell replacement. So, the way it improves functional recovery must be elucidated in future studies (Skop et al., 2019).

## Conclusion

One of the main factors that preclude the application of chitosan is its poor solubility and poor mechanical properties. However, this review summarizes the different strategies that have been used to overcome these conditions. The obtention of carboxymethylated, trimethylated, thiolated and other chitosan-grafted derivatives has increased the potential of this biopolymer, allowing for the elaboration of biomaterials that help to counteract neurological disorders. Nevertheless, there is still a lack of knowledge about the relation of the molecular changes and the acquired biological properties of these derivatives, especially within a heterogeneous landscape as the CNS. In this way, the authors suggest continuing with the exploration of grafting molecules that improve the biological properties of modified chitosan. For example, hydroxycinnamic acids have been studied by some of the authors and resulted in interesting bioconjugates for CNS applications.

Chitosan-based biomaterials have shown favorable projection. For drug and gene delivery purposes, chitosan nanoparticles have shown to be the most promising strategy due to its mucoadhesion and increased permeability that suits nose-to-brain applications. In this way, the degradation of the therapeutic molecules is reduced, and it also opens the door to less invasive and more effective administration routes. In order to achieve neuro-regeneration, the transplant of stem cells into chitosan-based vehicles gives an optimistic outlook. Strategies like the addition of neurotrophic factors or even the genetic modification of stem cells have successfully increased differentiation and viability. The observed functional recovery in different chitosan-based regenerative therapies encourages the exploration of new cell-scaffold-biomolecule configurations. Despite the wide variety of designed compositions and functions, many factors are implied in cell behavior and, until now, there is not a recipe to elaborate adequate chitosan-based biomaterials that fulfill all the requirements for neuro-repair and to transfer these strategies to clinical trials. Even so, the *in vitro* and *in vivo* studies carried out around the world are helping to understand the biological processes involved in neuro-repair and the effect of chitosan biomaterials on them. The authors suggest that future works with chitosan targeting the CNS must intermix the already suggested strategies and propose novel interdisciplinary approaches to attain translation into the clinical level.

## Author Contributions

DO-H, AC-A, JM-G, UG-P, and JM-D equally contributed to the literature search, writing and correcting of this review manuscript.

## Conflict of Interest

The authors declare that the research was conducted in the absence of any commercial or financial relationships that could be construed as a potential conflict of interest.
